# Spleen and peripheral blood immunopathology in an outbred model of adult-stage murine schistosomiasis

**DOI:** 10.3389/fimmu.2025.1527129

**Published:** 2025-05-01

**Authors:** Thomas T. Schulze, Andrew J. Neville, Evie G. Ehrhorn, Sarah A. Alsuleiman, Jonathan L. Vennerstrom, Paul H. Davis

**Affiliations:** ^1^ Department of Biology, University of Nebraska at Omaha, Omaha, NE, United States; ^2^ Department of Pathology, Microbiology, and Immunology, University of Nebraska Medical Center, Omaha, NE, United States; ^3^ Department of Pharmaceutical Sciences, University of Nebraska Medical Center, Omaha, NE, United States

**Keywords:** schistosomiasis, helminth, trematode, *Schistosoma mansoni*, cytokine profile, hematology, granulocytosis

## Abstract

Schistosomiasis, a parasitic disease caused by flatworms of genus *Schistosoma*, is a neglected tropical disease that causes significant morbidity in the developing world. Despite numerous efforts to eradicate the disease, the parasite remains a significant global burden, particularly within Sub-Saharan Africa. *Schistosoma* species possess an arsenal of potent mechanisms to defend against direct attack from host immune cells and a remarkable ability to modulate the host immune system through proximal and systemic modes that facilitate its stage-specific development, survival, and reproduction. Standardized animal models have been developed that serve as an important resource for dissecting parasite and host immunobiology and for drug and vaccine efficacy studies. However, a comprehensive understanding of the immune responses to *Schistosoma mansoni* in the standard outbred Swiss Webster mouse model is still lacking, particularly with the granulocyte composition of the spleen and the associated blood cytokine responses that occur during chronic infections. To continue characterization of this important secondary lymphoid tissue and the peripheral blood, we examined infected mouse spleens and additionally performed a detailed flow cytometric analysis of the splenic compartment from infected mice with specific attention to granulocytes and Th2-associated leukocytes. Peripheral blood from infected animals was used to evaluate a panel of Th1- and Th2-associated cytokines for comparison. Lastly, an analytical blood count and differential was also reported to provide a case study of late-stage chronic schistosomiasis. In mice infected with *S. mansoni*, we identified granulocytosis within the spleen including increased eosinophils, neutrophils, basophils, and mast cells. Additionally, ILC2s and dendritic cells were increased. The cytokine data suggests an IL33-independent mixed Th1/Th2 response with upregulation of granulocyte proliferative and recruitment factors. The late-stage chronic schistosomiasis case study identified blood neutrophilia and eosinophilia but with absent basophils. These data enhance our understanding of the complex immune response that occurs with schistosomiasis and may offer new insights to support therapeutic strategies against this important disease.

## Introduction

Schistosomiasis is a significant human disease caused by helminths of the genus *Schistosoma*. The disease is highly debilitating and typically associated with impoverished regions (1). To date, six *Schistosoma* species capable of infecting humans have been identified with most cases caused by *S. mansoni*, *S. haematobium*, and *S. japonicum* (1–3). Currently, schistosomiasis is thought to infect at least 200 million people with sub-Saharan Africa carrying the highest incidence (4, 5). Schistosomiasis is rarely fatal; however, the infected may suffer from chronic disease-associated morbidities (6). Recent efforts to estimate the global burden of disease highlight the challenges associated with estimating the true impacts of chronic diseases such as schistosomiasis (7). Likely contributors that hinder the control of human schistosomiasis include poor infrastructure development, less than ideal treatment options, and a lack of a vaccine (3, 8). Moreover, reliance on a limited range of and extended treatment timeframes of chemotherapies (i.e. praziquantel) has led to suspected drug resistance in some species (9). Consequently, there is a renewed interest in single-dose therapies, particularly those that clear the juvenile and adult stages of infection, such as those reported in Gardner et al. (10), and the recently optimized aryl hydantoin compound series (11).

Helminth parasites have developed an impressive persistence strategy that includes both immune evasion and remodeling, enabling them to parasitize the host for years and to facilitate egg release (12). Accordingly, the immunobiology of schistosomiasis is complex and includes a temporal lifecycle-associated immune profile that shifts during the transition from initial infection to mature egg-producing worm pairs (13). Additionally, the immune environment is multifactorial where the worms, eggs, and host immune response are all known to participate in unique and competing immune-modulating behaviors. An incomplete understanding of schistosomiasis immunology and pathogenesis has been a barrier to breakthroughs that are urgently needed to design more effective treatments and to identify new opportunities to exploit the parasite’s immune modulation strategies. During the initial acute infection (typically 4-5 weeks post-infection with cercariae), the developing juvenile worms trigger a mixed immune response composed of Th1- and Th2-associated factors (13). At approximately 5-6 weeks of development, egg production begins, and the host immune response is pushed further toward a Th2-dominant environment (13, 14). The Th2 environment is characterized by elevated IL-4, IL-5, and IL-13, and is accompanied by elevated granulocytes such as eosinophils (15, 16). Notably, many granulocyte effector molecules (e.g. eosinophil peroxidase, neutrophil elastase) have shown potent effects on adult worms and schistosomula *in vitro* (17–19), yet worms successfully persist inside hosts for years despite continual infection-associated granulocytosis (16). As the disease progresses to the chronic stage, the Th2 environment can diminish as IL-10 is secreted by regulatory lymphocytes which are thought to control disease severity (15). Notably, the tug-and-pull between Th1 and Th2 during infection must be balanced as hyperpolarization (in either direction) was demonstrated to be lethal in animal models (15). Indeed, understanding the immunology and pathogenesis of schistosomiasis may be key as many treatments, including praziquantel, show an immune requirement, where host immune components appear to be necessary for effective worm burden reduction (20–23).

Murine schistosomiasis has become the preferred animal model for human schistosomiasis and has been used for various studies, particularly for immunology and therapeutic development (24, 25). However, maintenance of the intermediate parasite life stages and optimization of animal infection methods remain challenging and require training and specialty facilities (26). To address these challenges, the NIH-supported Schistosomiasis Resource Center maintains the parasite life cycle for multiple *Schistosoma* species (e.g. *mansoni*, *japonicum*, and *haematobium*) and provides standardized infected rodent models for end-users to utilize for studies. While these animal models have been used to dissect many aspects of immunobiology, we still lack a full understanding of innate immune and tissue-specific granulocyte populations, particularly within secondary lymphoid tissues (e.g. spleen) which contribute to the collective immune response and may participate in antigen presentation (27). The objective of this study was to utilize this rodent model to characterize how the splenic compartment and peripheral blood cytokine environment change in response to chronic *S. mansoni* infection with a focus on Th2 effector granulocytes and the associated innate lymphoid cells (i.e. ILC2) which are thought to participate in the immune response.

Here we investigated the outbred Swiss Webster (CFW) *S. mansoni* mouse model, performing a detailed evaluation of the infected spleen, including flow cytometric analysis of granulocyte composition among splenocytes. We observed elevated granulocyte populations and increased Th2-associated innate cells, including eosinophils, neutrophils, basophils, mast cells, and type 2 innate lymphoid cells (ILC2s). Cytokine analysis of infected animal peripheral blood suggests an IL33-independent mixed Th1/Th2 cytokine response with hallmarks of inflammation and immune regulation, in agreement with previous observations (28–30). Lastly, in a single mouse case study that survived and presented a very late-stage chronic schistosomiasis infection (19 weeks post-infection), blood hematology parameters, and a complete leukocyte differential indicated neutrophilia and eosinophilia, but absent of basophils.

## Materials and methods

### Establishing adult-stage and chronic schistosomiasis in mice

*S. mansoni* infected Swiss Webster CFW female mice were obtained from the Schistosomiasis Resource Center (Rockville, MD). Mice were purchased from either Taconic or Charles River Laboratories. Infections were performed percutaneously by tail exposures to freshly hatched cercariae (approximately 200 per mouse: average 190, range 182-192 cercariae) when mice were 5-6 weeks old. All infections were performed with *S. mansoni* strain NMRI (BEI Resources, NR-21963).

### Isolation of spleen for phenotypic analysis

Three uninfected and infected mice were asphyxiated with CO_2_ prior to tissue collection. Spleens were harvested immediately and maintained in 1X HBSS without calcium, magnesium, and phenol red (Corning, cat# 21022CV) at 4°C until images were taken and masses measured using an analytical balance. Spleen samples were stored <4 h before inspection. Statistical analysis (unpaired t-test) and graphing were performed with GraphPad PRISM ver. 10.2.3. The bar graphs presents the mean and standard error of the means (SEM) (error bars) for each group (n=3 mice per group). The infected animals used in these studies were 13 weeks old and 7 weeks post-infection (PI), and uninfected mice were age-matched.

### Flow cytometric analysis of splenocytes during adult-stage schistosomiasis

The infected mice used in these studies were 13 weeks old and 7 weeks post-infection. Uninfected mice were age-matched to infected mice. The method and precise protocol used for preparation of splenocytes was previously optimized and published by our lab (31). Briefly, three outbred Swiss Webster female mouse spleens were pooled for both the uninfected and infected samples that were used for the flow cytometry studies. Splenocytes from either infected or uninfected mice were used for each group’s FMOs and unstained controls to account for potential differences in cell autofluorescence and other possible variations between the infected and uninfected splenocyte samples. Bead compensation controls (MACS^®^ Comp Bead Kit, anti-REA, Miltenyi Biotec, cat.# 130-104-693) were run in parallel for each flow cytometry acquisition run sample to account for instrument drift and to construct the spectral matrices. An LSRFortessa™ X-50 (BD Biosciences) flow cytometer instrument was used for data acquisition and the analyses and plots were executed with FlowJo™ v10.10.0 Software (BD Life Sciences). Annotated figures were prepared with Adobe Illustrator ver. 28.7.0 (Beta). Each marker evaluated had its own associated fluorescent minus one (FMO) control which was used for strict gating of positive/negative populations. The gating strategy is illustrated in the respective figures.

### Peripheral blood cytokine analysis

Uninfected and infected Swiss Webster CFW females (n=8 per group) were age-matched within 1 week (approximately 7 weeks post-infection, approximately 200 cercariae percutaneously) at the time of sample collection. 24 hours before cardiac blood collection, mice received a common drug solvent (90/3/7% of water, ethanol, and Tween^®^80, respectively). The solvent was filter-sterilized using a 0.22 µm PES membrane (MilliporeSigma™, cat# SCGP00525), and orally (PO) administered volume of 4.0 µL/gram of mouse weight via an 18G oral gavage (SAI Infusion Technologies, cat# FN18-38M). This solvent was used as a vehicle comparator, commonly used as a drug vehicle solvent in drug discovery efforts using the *S. mansoni* outbred mouse model. 24 hours post-administration of solvent, mice were euthanized by CO_2_ asphyxiation and peripheral blood was isolated via cardiac puncture using a 1 mL syringe (Easy Glide Sterile Syringe Luer Lock; EGLL1ML-100) with attached 23-gauge needles (BD, catalog # 305143). Blood was immediately transferred to MiniCollect^®^ TUBE 0.8 ml CAT Serum Separator (Greiner, catalog # 450472) tubes and allowed to clot at room temperature for 30 minutes. Samples were spun at 2,000 x g for 10 minutes at room temperature and the supernatant containing serum was aliquoted in 1.5 mL low-retention microcentrifuge tubes (Thermo Scientific, cat# 3451) and stored at -20 °C until analysis. Serum samples were diluted 1:4 in Diluent 41 reagent (Meso Scale Diagnostic Services, cat# R50AH-1) and assayed using a 10 analyte Meso Scale U-PLEX Custom Biomarker Group 1 (mouse) Multiplex Assay Kit (Meso Scale Diagnostics, cat# K15069L-1) following the manufacturer’s instructions. A Meso Scale QuickPlex SQ 120 model 1300 instrument was used to measure electrochemiluminescene (ECL) signals and the raw data was initially prepared with the Meso Scale WorkBench 4 Software version 4.0.13 (Meso Scale Diagnostics). Data was transformed in Microsoft Excel version 2.411 (Microsoft, USA) and was analyzed and graphed with GraphPad Prism ver. 10.2.3 and statistical significance was determined by a two-tailed unpaired t-test. Cytokine concentrations (pg/mL) are presented as the mean ± standard error of the mean (SEM).

### Analytical complete blood count and differential

Reference uninfected Swiss Webster CFW female mice (n=7) were solvent-treated as above and euthanized with CO_2_ asphyxiation prior to immediate blood collection. Peripheral blood was obtained through cardiac punctures with 23-gauge needles (BD, catalog # 305143) attached to S-Monovette^®^ K_3_EDTA tubes (Sarstedt, catalog # 06.1664.100) and immediately inverted 8-10 times and stored at 4 °C. Samples were shipped to IDEXX facilities after preparation (same day) for processing the following morning. IDEXX test code 63160: Comprehensive CBC with MPV, RDW and Retic Hgb was performed. The infected mouse (n=1) was approx. 6 months old at time of sampling, and 19 weeks post-infection.

## Results

### Spleens from mice with schistosomiasis show splenomegaly and malformations

**Figure 1:**
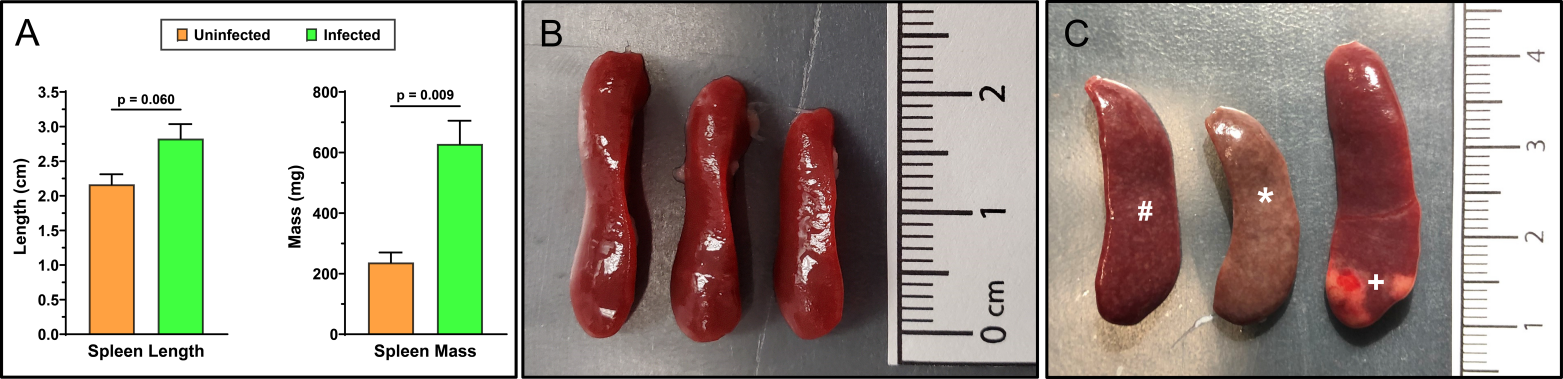
Spleens of adult-stage *S. mansoni* infected mice have pronounced splenomegaly, discoloration, and malformations. Mean spleen masses and lengths **(A)** were obtained from female Swiss Webster mice that were either uninfected **(B)** or 7 weeks post-infected percutaneously with ~200 *Schistosoma mansoni* cercariae **(C)**. Spleens were visually inspected and measured for masses and lengths to detect splenomegaly (n=3 mice/group). Mean infected masses and lengths show clear and significant splenomegaly that is associated with chronic schistosomiasis. Annotated discolorations (*), varied malformations, and some larger regions resembling granulomas or fibrotic tissue (+) are noticeable in the infected spleens **(C)**. Additionally, a consistent spotted phenotype (#) was observed across all infected spleens but absent from uninfected spleens. Infected and uninfected mice were age-matched. Data was analyzed and graphed with GraphPad Prism ver. 10.2.3 and statistical significance was determined with a two-tailed unpaired t-test. Data is presented as the group mean ± standard error of the mean (SEM).

Spleens obtained from infected animals were first examined macroscopically for phenotypic differences compared to normal, uninfected reference spleens. The spleens from infected animals showed increases in both length and mass and obvious discolorations consistent with splenomegaly (**Figure 1**). The increased spleen masses were statistically significant (n=3; p=0.009) whereas increases in length were near significance (n=3; p=0.060). Notably, discoloration and a spotted phenotype was observed in the infected spleens (**Figure 1C**) with one organ showing severe discoloration resembling a large granuloma or tissue fibrosis (32). In comparison to organs obtained from mice infected with *S. japonicum*, the spleen malformations appear less heterogenous or more evenly distributed (32).

### Flow cytometric characterization of splenocytes from adult-stage schistosomiasis

To dissect the cellular composition of the infected spleens vs. uninfected controls, an optimized flow cytometry panel was utilized with modifications (31) to examine the granulocyte compartment of total mouse splenocytes. Splenocyte samples were prepared by pooling whole spleen from multiple donors (n=3 spleens per condition) prior to generating splenocyte suspensions, to obtain a representative sample. Notably, the protocol used for preparation of cell suspensions was previously optimized in our lab to maximize granulocyte recovery and reduce cellular activation during preparation (31). Granulocytes are known to be sensitive, short-lived and easily activated during preparation based on the isolation methods used (e.g. density gradients (33); moreover, the composition of processing buffers (e.g. BSA or fetal serum) can significantly affect cell recovery (31, 34, 35).

**Figure 2:**
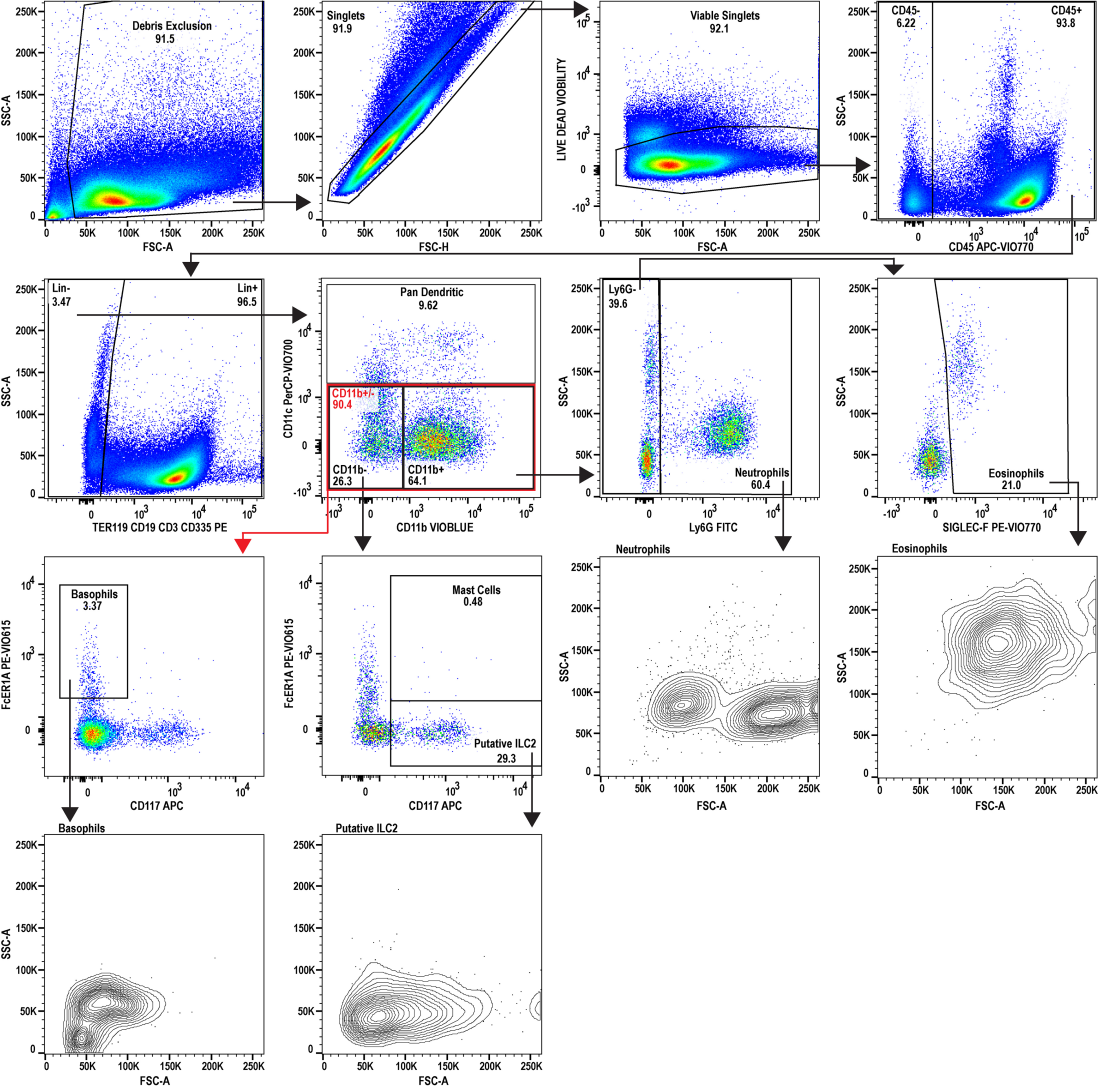
Flow cytometric analysis of uninfected reference splenocytes. A 9-color flow cytometry panel was utilized to profile the splenic immune cells and to specifically inspect the granulocyte lineage. A dump gate is included to filter out abundant lymphocytes (CD3^+^, CD19^+^, CD335^+^) and Ter119^+^ cells which are denoted as Lin^+^. Gating was performed to isolate eosinophils, neutrophils, basophils, and mast cells. Additionally, type 2 innate lymphocytes (ILC2) and dendritic cells were also inspected. FSC-A and SSC-A plots are also provided for the granulocytes for phenotyping activation state and to examine heterogeneity. This uninfected sample serves as the reference for comparing to the infected sample (see **Figure 3**). The splenocytes used for analysis were prepared by pooling spleens from three individual donor mice (n=3, Swiss Webster CFW females). Data analysis was performed with FlowJo™ v10.10.0 Software (BD Life Sciences).

**Figure 3:**
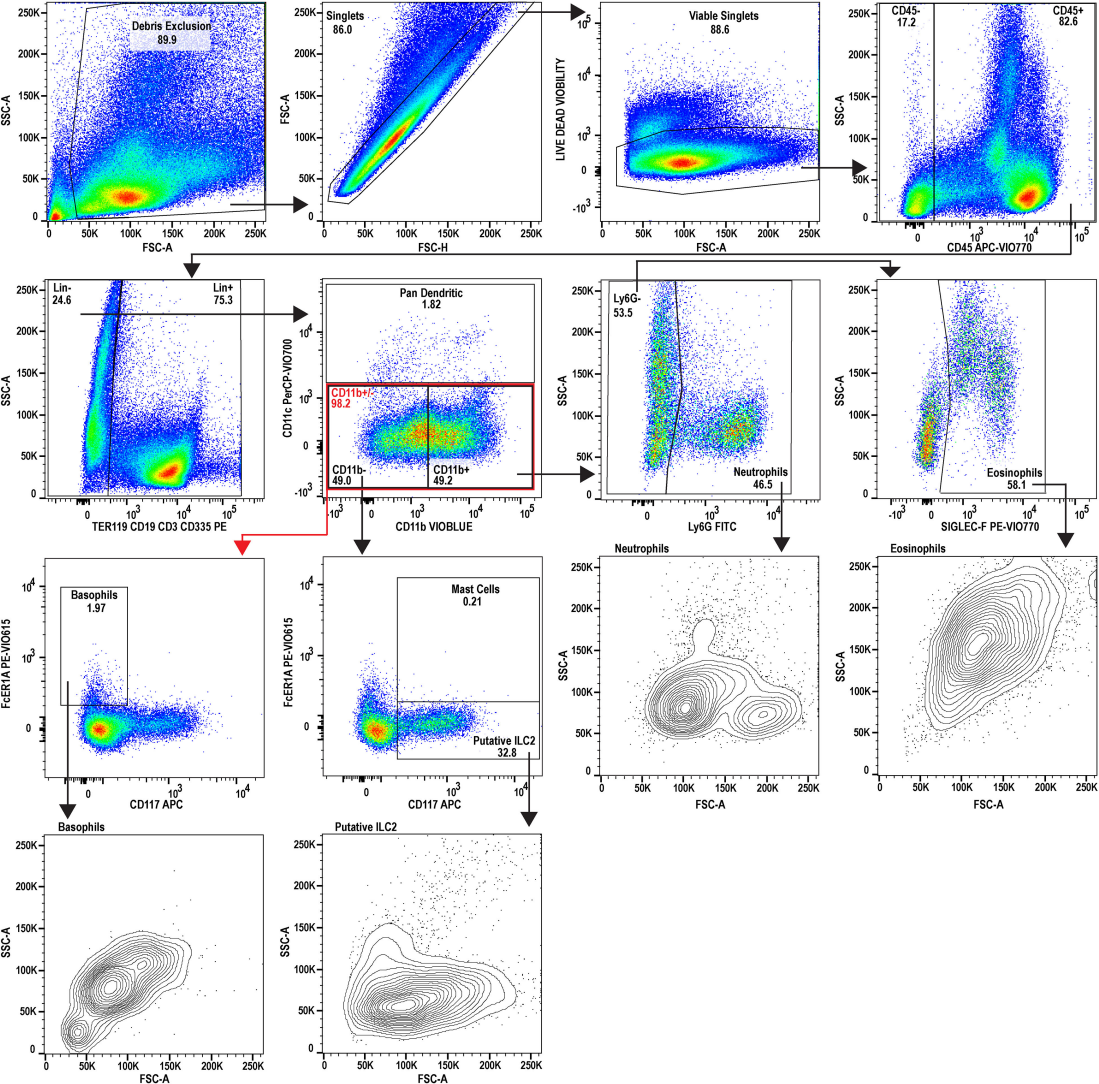
Flow cytometric analysis of splenocytes during adult-stage schistosomiasis. A 9-color flow cytometry panel was utilized to profile the splenic immune cells from infected animals, to specifically inspect the granulocyte lineage, and gated identically to **Figure 2**. FSC-A and SSC-A plots for the granulocytes examined suggest a stimulated or activated cellular phenotype supported by increases to SSC-A and increased heterogeneity in neutrophils. The putative splenic ILC2 population additionally appears to be expanded during schistosomiasis. The splenocytes used for analysis were prepared by pooling spleens from three individual donor mice infected with *Schistosoma mansoni* (n=3, Swiss Webster CFW females, 7 weeks post-infection with approx. 200 cercariae percutaneous). Data analysis was performed with FlowJo™ v10.10.0 Software (BD Life Sciences).

For relative quantification of the cell subsets, the results are calculated with FlowJo™ software and reported using both percent of intact single-cells and as a percent of CD45^+^ cells (**Table 1**), where CD45 serves as a general marker of leukocytes. The gating strategies and resulting cell populations are shown in for the uninfected reference (**Figure 2**) and infected (**Figure 3**) samples. A large increase in the CD45^-^ subset, relative to the overall sample/cell population, was seen in the infected (14.31%) versus uninfected (2.23%) which is shown in **Figures 2**, **3** (panel SSC-A vs. CD45). This may be due in part to altered hematopoiesis that is associated with murine schistosomiasis and can cause increased events that are CD45^-^ and are likely Ter119^+^ (e.g. erythrocytes from altered splenic erythropoiesis) (36, 37). Essentially, this might indicate expansion of the erythroid lineage in the spleen that reduces the proportion of cells that are CD45^+^.

**Table 1:** Flow cytometry analysis of splenocytes in uninfected reference vs. adult-stage schistosomiasis mice.

Cell Population	Gating Strategy	Reference Uninfected (% of CD45+)	Infected (% of CD45+), Change vs. Reference	Reference Uninfected (% of Single Cells)	Infected (% of Single Cells), Change vs. Reference
Neutrophil	CD11c^-^CD11b^+^Ly6G^+^	1.34	5.63, 4.20x	1.16	4.12, 3.55x
Pan-Dendritic/ Macrophage	CD11c^+^	0.33	0.45, 1.36x	0.29	0.33, 1.14x
Basophil	CD11c^-^Ly6G^-^FcϵR1α^+^CD117^-^	0.11	0.48, 4.36x	0.091	0.35, 2.95x
Eosinophil	CD11c^-^CD11b^+^Ly6G^-^SiglecF^+^	0.18	3.77, 20.9x	0.16	2.76, 17.3x
Mast	CD11c^-^CD11b^-^Ly6G^-^ FcϵR1α^+^CD117^+^	4.38E-3	0.025, 5.71x	3.78E-3	0.019, 5.03x
ILC2, putative	CD11c^-^CD11b^-^Ly6G^-^SiglecF^-^CD117^+^	0.27	3.95, 14.6x	0.23	2.89, 12.6x

The splenocyte samples visualized in the representative flow cytometry panels are from a single experiment (n=1) and were generated from pooled spleen samples (n=3 animals for uninfected and infected) to generate a representative sample for the outbred background (female Swiss Webster CFW) and infected condition. The preparation procedure was adapted from (31). Splenocytes were analyzed via multicolor (9-color) flow cytometry to measure changes to relevant granulocytes and innate lymphocytes to profile the splenic composition during murine chronic schistosomiasis *mansoni*. Cell populations shown here are reported as either a percentage of single intact cells or a percentage of CD45^+^ for comparison and were calculated in FlowJo software version 10.10.0. All populations included here are Lin^-^CD45^+^; Lin=Ter119^+^CD3^+^CD19^+^CD335^+^. The ILC2 population is putative and may contain additional subtypes. The pan-dendritic/macrophage gate may also contain monocytes, which can express both CD11c and CD11b at varying levels.

**Figure 4:**
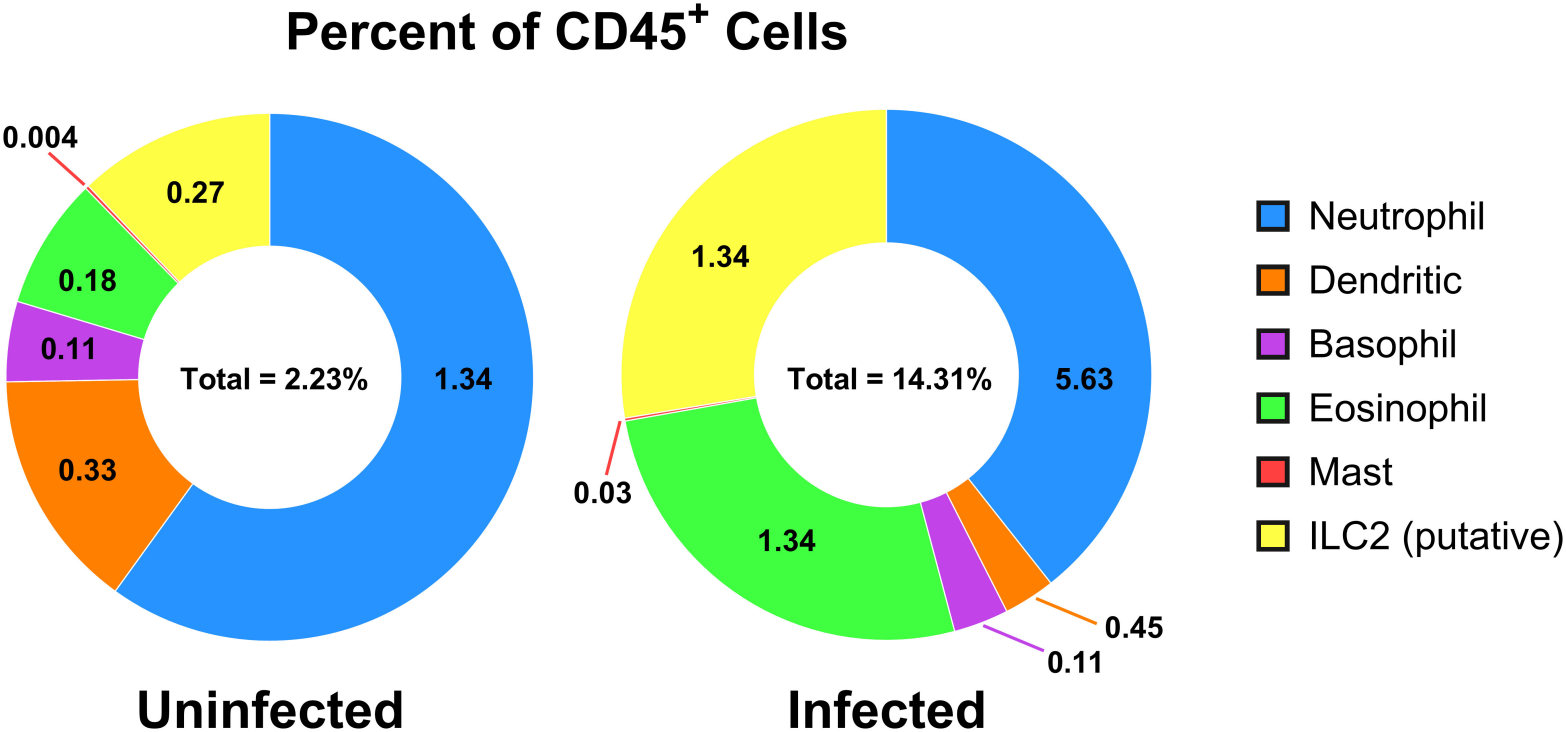
Cell populations of CD45^+^ cells in uninfected versus adult*-*stage schistosomiasis-infected spleens. Presented is a summary figure of the spleen flow cytometry data presented in **Table 1**, corresponding to the flow cytometry analyses of the uninfected (**Figure 2**) and infected (**Figure 3**) mouse spleens. The donut figure displays each cell type as the percentage of all CD45^+^ cells. Data analysis was performed with FlowJo™ v10.10.0 Software (BD Life Sciences) and the figure was generated using GraphPad Prism version 10.2.3.

Further analysis of the infected mouse splenocytes shows expanded populations of granulocytes including neutrophils, basophils, eosinophils, and mast cells (**Table 1**; **Figure 4**). Spleen-derived eosinophils showed the largest increase (20.9x) in this dataset followed by putative ILC2s (14.6x increase). Neutrophils, basophils and eosinophils all showed a marked increase ranging between 4-5x (% of CD45^+^). Additionally, the granulocyte populations isolated in the infected mice (**Figure 3**) showed increased SSC-A profiles, indicating increased granularity and likely signifying an activated or stimulated phenotype that is differentiated from uninfected mice (**Figure 2**) (38, 39). Dendritic cells have previously been shown to play a role in the induction of the Th2 response in response to eggs (40). Pan-dendritic cells (Lin^-^CD45**^+^** CD11c^+^) showed an approximate 1.36x increase in their abundance and showed variable CD11b expression that was present in both the uninfected reference (**Figure 2**) and infected samples (**Figure 3**). Notably, the gating strategies utilized here can be tissue specific. For example, spleen derived eosinophils are Lin^-^CD45**^+^** CD11b**^+^** CD11c^-^Ly6G^-^SiglecF**^+^**; however, this can differ by tissue and mouse strain (41). Bone marrow and lung derived eosinophils have been shown to contain a CD11c^low^ population which might render this gating strategy ineffective in those tissues (42).

Innate lymphoid cells (ILCs) lack surface markers commonly found on T and B cells and are known to be tissue-resident and immune regulatory (43). ILCs are now understood to sustain immune responses, react early compared to adaptive immune counterparts, and contain subgroups such as ILC1, 2, and 3 corresponding to Th1, Th2, and Th17 associated cells (43). The ILC2 subset has been previously identified in mouse bone marrow, lung, fat, gut, and skin tissues with an apparent tissue-specific transcriptome (44). ILC2s are thought to be tissue-resident and long-lived but may move between tissues (45) and are typically associated with the production of IL-5, IL-9, and IL-13 (46). As ILC2s are thought to be principally associated with Th2 immune responses that are induced during schistosomiasis (45, 47), they were assessed in the infected spleen. Notably, as the ILC cell surface markers often vary by tissue of residence, and have yet to be fully characterized, these should be considered putative ILC2s which may contain other subtypes and may have migrated from other tissues. We identified a minor population in the spleen (<1% in the reference; identified as Lin^-^CD45**^+^** CD11b**^-^** CD11c^-^Ly6G^-^SiglecF^-^FcERIα^-^CD117^+^) that expanded 14.6x (% of CD45^+^) during chronic schistosomiasis (**Table 1**; **Figure 4**).

### Cytokine profiling during adult-stage schistosomiasis indicates a mixed Th1/Th2 phenotype

**Figure 5:**
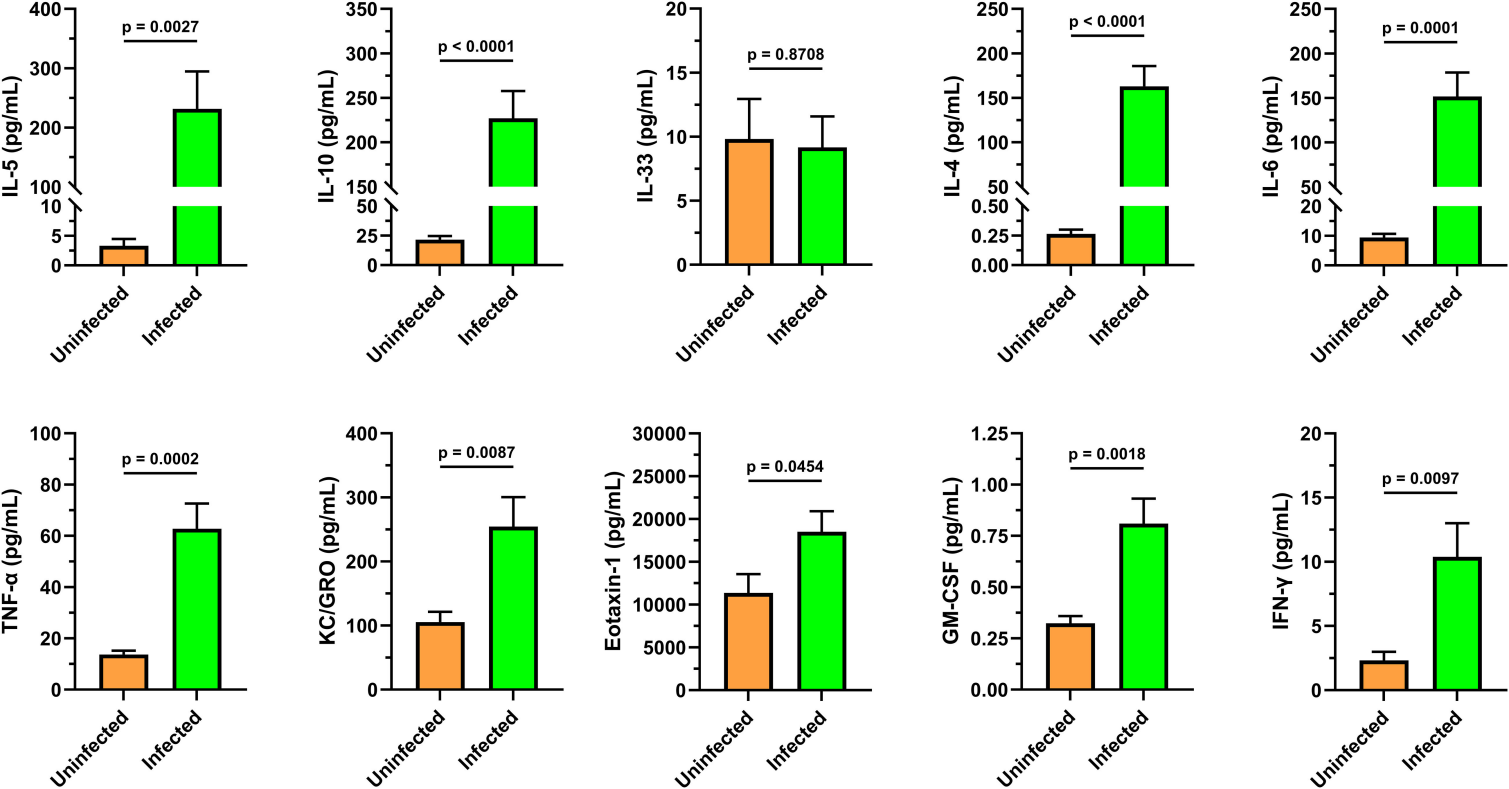
Cytokine analysis of infected animal cardiac blood identifies mixed Th1/Th2 immune response. A custom MesoScale U-PLEX assay was designed to assay a panel of 10 cytokines including markers that associate with Th1 and Th2 immunity to characterize the immune environment to compare to the spleen flow cytometry (**Figures 3**, **4**) and blood analytical datasets (**Tables 2**, **3**). The cytokine signals indicate a complex immune response characterized by Th1 and Th2 cytokines that appear to be independent of IL33/St2 signaling. Granulocyte-associated factors such as GM-CSF, Eotaxin-1, and KC/GRO all showed significant upregulation. These serum samples were collected from uninfected (n=8) and infected (n=8) female CFW Swiss Webster mice that were approximately 7 weeks post-infection with *S. mansoni* (approx. 200 cercariae percutaneous). All samples were within their respective lower (LLOD) and upper (ULOD) limits of detection, except for the IL-4 levels in the uninfected group were slightly lower than the 0.56 pg/mL LLOD of IL-4. Data was analyzed and graphed with GraphPad Prism ver. 10.2.3 and statistical significance was determined with a two-tailed unpaired t-test. Data is presented as the group mean ± standard error of the mean (SEM).

It is now understood that cytokine profiles differ by stage of infection (i.e. weeks post-infection), the species/strain of *Schistosoma*, magnitude of infection, and the background strain of mice (48). During initial infections, *Schistosoma* species trigger a Th1 immune response that transitions over time to a Th2 environment (49). These environments typically include elevated IL-4, IL-5 and IL-13 (50, 51). To continue the cytokine characterization for this standardized model of *S. mansoni*, we generated a blood cytokine profile to compare to the splenocyte datasets. A custom U-plex MesoScale plate was designed with a panel of Th1 and Th2 cytokines/chemokines, including those with known responses, and a subset of less characterized cytokines that are granulocyte-associated (e.g. Eotaxin-1, GM-CSF, KC/GRO). CFW female mice (n=8) were utilized for each group to compare uninfected vs. adult-stage schistosomiasis (approximately 7 weeks post-infection). The means and standard error of the means (SEM) for each analyte are displayed in **Figure 5**. Notably, and with the exception of IL-33, all cytokines/chemokines measured were significantly increased (p<0.05) indicating a mixed proinflammatory phenotype including both Th1 and Th2-associated cytokines. IL-33 is considered an alarmin associated with cell damage that can function as a driver of Th2 responses by stimulating the release of IL-5 and IL-13 (52). However, it has been shown in *S. mansoni*-infected animals that IL-33 and its associated ST2 pathway are not essential for the Th2 polarization that occurs during disease, which is supported by this dataset (28).

### Late-stage chronic schistosomiasis hematology and comprehensive differential case study

A single mouse (Swiss Webster CFW female) with late-stage chronic schistosomiasis (19 weeks post-infection) was included in an expanded complete blood count and differential to examine how the blood compartment is altered, and to compare to the changes measured within the spleen (approximately 7 weeks post-infection). The blood data results are available in **Table 2** and the leukocyte differential is included in **Table 3**. Consistent with the splenic data, granulocytes in the blood compartment were increased including neutrophils and eosinophils, with eosinophils showing the largest increases (31x vs. reference, **Table 3**). Interestingly, while basophils showed increased splenic residence (**Table 1**), they were undetectable in the peripheral blood of the infected animal, yet the reference animals showed a small population in circulation (11/µL on average, **Table 3**). The blood analysis (**Table 2**) similarly showed elevated absolute counts of WBCs in the chronically infected mouse (25.3 K/μL) versus uninfected mice (7.2 K/μL) and slight changes to parameters such as HGB, HCT were detected, in addition to others.

**Table 2:** Schistosomiasis hematology case study, late-stage chronic infection.

CBC Parameters	Reference Uninfected, n=7, mean (SD)	Chronically Infected, n=1, 19 Weeks Post-Infection
WBC (K/µL)	7.2 (1.8)	25.3
RBC (M/µL)	10.06 (0.41)	9.21
HGB (g/dL)	15.2 (0.4)	13.8
HCT (%)	47.2 (1.6)	44.7
MCV (fL)	47 (2)	49
Red Cell Distribution Width (%)	23.3 (0.8)	22.4
MCH (pg)	15.1 (0.4)	15
MCHC (g/dL)	32.2 (0.4)	30.9
Platelet estimate	adequate	adequate
Platelet count (K/µL)	891 (61)	775
MPV (fL)	7.0 (0.2)	7.5
RBC Morphology		
Manual Retic (%)	5.5 (1.0)	7.2
Reticulocyte (%)	4.3 (0.7)	3.7
Absolute Retic based on Manual Retic (%)	547 (92)	663
Absolute Reticulocyte (K/µL)	436 (70)	341
Reticulocyte Hemoglobin Content (pg)	18.3 (0.5)	18
Nucleated RBC (/100 WBC)	none seen	none seen
Polychromasia	moderate^a^	moderate
Anisocytosis	slight	slight
Poikilocytosis	none seen	none seen
Heinz bodies	none seen	none seen

a For the inspection of samples for polychromasia in the uninfected mice, 1 animal from an n=7 showed “slight” and the remaining 6 showed “moderate”.

A single animal case study (Swiss Webster CFW female) with late-stage chronic schistosomiasis (19 weeks PI) was used to isolate peripheral blood via the cardiac route and was analyzed and provided by IDEXX BioAnalytics with the Comprehensive CBC with MPV, RDW, and Retic Hgb; test code 63160. The general CBC, hematological parameters, and detailed investigations into the erythroid lineage and cell examinations are presented. Late-stage schistosomiasis shows alterations to WBCs, platelets, RBCs, and reticulocytes.

**Table 3:** Schistosomiasis comprehensive leukocyte differential case study, late-stage chronic infection.

Relative Leukocyte Counts	Reference Uninfected, n=7, mean (SD)	Chronically Infected, n=1, 19 Weeks Post-Infection
Neutrophil (%)	8.3 (1.5)	10
Lymphocytes (%)	86.2 (2.6)	69
Band (%)	none seen	none seen
Eosinophil (%)	1.5 (0.8)	13
Monocyte (%)	3.9 (1.3)	8
Basophil (%)	0.2 (0.1)	0
Metamyelocyte (%)	none seen	none seen
Myelocyte (%)	none seen	none seen
Promyelocyte (%)	none seen	none seen
Unclassified (%)	none seen	none seen
Absolute Counts		
Unclassified (/µL)	none seen	none seen
Neutrophil (/µL)	580 (133)	2530
Band (/µL)	none seen	none seen
Eosinophil (/µL)	106 (67)	3289
Basophil (/µL)	11 (4)	0
Monocyte (/µL)	286 (132)	2024
Lymphocyte (/µL)	6174 (1600)	17457
Metamyelocyte (/µL)	none seen	none seen
Myelocyte (/µL)	none seen	none seen
Promyelocyte (/µL)	none seen	none seen

A single animal case study (Swiss Webster CFW female) with late-stage chronic schistosomiasis (19 weeks PI) was used to isolate peripheral blood via the cardiac route and was analyzed and provided by IDEXX BioAnalytics with the Comprehensive CBC with MPV, RDW and Retic Hgb; test code 63160. The panel includes a comprehensive leukocyte differential with relative (%) and absolute counts. Leukocyte subtypes and progenitors are also included. Marked increases in most blood leukocytes were identified, however basophils were absent from circulation.

## Discussion

Schistosomiasis remains a significant challenge in the developing world and a better understanding of the complex immune pathology may lead to the design of more effective therapeutics and vaccines. Despite documented susceptibilities to host granulocytes, which increase during infections and display activated or primed cellular phenotypes, *Schistosoma* species persist inside hosts for years in humans (53). In this work we provide a brief phenotypic analysis of infected mice spleens, a detailed inspection of the granulocyte and innate lymphocyte content of the mouse splenic compartment and assess a panel of Th1- and Th2-associated cytokines in the peripheral blood during adult-stage schistosomiasis, which can vary widely by tissues and infection stage (13). Lastly, we present a case study that includes an analytical blood count and leukocyte differential comparing uninfected mice to a late-stage chronically infected mouse. In the context of schistosomiasis, host tissues such as the liver are relatively well studied where secondary lymphoid tissues, such as the spleen, have been neglected. Immunophenotyping and characterization studies such as these are useful for building a comprehensive understanding of the infected host immune environments and response to disease that can vary by *Schistosoma* species and the host/strain utilized (48).

The gross pathology observed in the spleen (**Figure 1**) are consistent with those that has been published in the literature to date (32). Egg deposition within spleens is uncommon with *Schistosoma* species but has been observed with species such as *S. japonicum* in C57BL/6 mice where deposition was observed to be common, but granulomas were less frequent (32). Notably, mice that did present with granulomas appeared to have changes to lymphoid follicles and enhanced humoral responses, indicated by increased IL-4, IL-5, and IL-10 in splenocyte supernatants (32). Case studies of eggs deposits in human spleens with *S. mansoni* have emerged more recently and it remains unclear if these are representative (54). These findings are relevant to human medicine as atypical egg deposits can present with symptoms that are misdiagnosed as different conditions, confusing medical practitioners, and resulting in contraindicated procedures or treatments (54, 55). In future studies, it would be useful to systematically evaluate egg burden with this model of Swiss Webster (CFW) mice, using similar methods that are used to harvest eggs from infected mice livers (i.e. homogenization followed by sieves), described in (56) to comprehensively investigate the frequency of egg deposition in mouse spleens, specifically for *S. mansoni*. It remains possible that these deposits are present but less obvious as they are typically not evaluated in the spleen.

The analysis of splenocytes and peripheral blood identified marked eosinophilia which is characteristic of *S. mansoni* (57). This coincides with (and is likely maintained by) elevated serum IL-5 measured in the cytokine data [**Figure 5**; (58)]. The splenic eosinophils and neutrophils in infected mice show an activated and/or differentiated phenotype indicated by an increased SSC-A, and an increase in the apparent neutrophil heterogeneity (**Figure 3**, neutrophil and eosinophil SSC-A vs. FSC-A) that are known to occur in a Th1/Th2 cytokine environment, in addition to density changes (59).

Mast cells have been shown to recruit to liver granulomas and schistosomula in primates; however, they remain understudied in the context of murine and human schistosomiasis (60, 61). Moreover, mast cell effectors may be necessary for cytotoxic responses to schistosomula (62). Previous studies with schistosomiasis identified increased colony-forming cells for mast cells and granulocyte-macrophages from bone marrow and splenic origin that correlated with increased transcription of IL-3 and IL-9 (63). We build upon these findings (comparing C57BL/6 to Swiss Webster CFW) and demonstrate the spleens of infected mice do appear to show an increased mast cell population (+5.71x as a %CD45^+^) that remains quite small in both the uninfected and infected spleens. However, it is gated with relatively high resolution as Lin^-^CD45^+^CD11b^-^CD11c^-^Ly6G^-^SiglecF^-^FcϵR1α^+^CD117^+^ supporting the population as mast cells (64–66).

There is an interesting link between chronic schistosomiasis and basophils where egg antigen exposure has been shown to trigger release of IL-4 and IL-13 (67) and basophils appear to be necessary for parasite egg-associated granuloma formation (68). However, some debate remains on which granulocyte is the dominant source of IL-4 (59). The *Schistosoma* egg antigen (SEA) was previously shown to be a principal driver of the Th2 immune response and is likely a key modulator driving these cytokine responses observed in blood (69). Indeed, Th2 polarization is well documented with murine and human schistosomiasis and appears to occur at the onset of egg production from adult worms (70). Our results support increased splenic basophils (Lin^-^CD45^+^CD11b^+^CD11c^-^Ly6G^-^SiglecF^-^FcϵR1α^+^CD117**^-^**) that appear to be absent in the peripheral blood of a case study with late-stage chronic schistosomiasis. Notably, the granulocytes gated here are gated on CD11b^+^. However, there exists a Lin^-^CD45^+^SiglecF^+^ cell population that is CD11b^-/low^ which appears to also expand during schistosomiasis (data not shown), possibly encompassing a progenitor population within the eosinophil or myeloid lineage. The neutrophil populations quantified here (**Table 1**) had no significant CD11b^-^ population (not shown).

In addition to the Th2-associated granulocytes, type-2 innate lymphoid cells (ILC2s) were also considered. It has been well established that the ILC2 subset is defined by GATA3 transcription factor dependence and produces type 2 cytokines (71). Moreover, IL-33, IL-25, and TSLP appear to be key activators of ILC2s in both mice and humans and are thought to be epithelial derived (72, 73). Based on the cytokine dataset from peripheral blood (**Figure 5**), we have identified a convincing expansion of a putative ILC2 population in the spleen that appears to be independent of IL-33. The data presented here supports previous reports that link schistosomiasis with induction of ILC2s (47). Notably, the measurement of innate lymphoid cells via flow cytometry is continually developing and markers can vary by mouse strain and tissue of analysis, and splenic ILC2s are less characterized versus those of lung and liver (74). Based on the gating strategy used here, this putative ILC2 population may also contain LTi cells that can be Lin^-^CD45^+^CD11b^-^CD11c^-^Ly6G^-^SiglecF^-^FcϵR1α^-^CD117^+^; yet this is not fully understood (71, 73). To further refine these subsets, the ILC2 population could additionally be gated for CD5^+^ to remove any residual T cells (75). Furthermore, to increase confidence in this population, ST2/IL-33R (73) or transcription factors GATA3_+_ and/or RORγt^−^ can be included (74).

The lack of significant change in IL-33 levels, despite a robust Th2 cytokine and cellular response, provides an interesting avenue that can be explored further; previous reports have shown that IL-33 appears to be dispensable for *S. mansoni* maturation and causes insignificant effects on egg abundance (29, 76). However, IL-33’s importance may differ by *Schistosoma* species, where *S. japonicum* infection reports identified increased serum IL-33 which corresponds to egg production suggesting it may be induced by eggs (77, 78). Indeed, parasite eggs are thought to be a primary driver of the Th2 immune responses observed across *Schistosoma* species, as oviposition timing correlates with cytokine production (79). Reports examining this effect in *S. mansoni* identified negligible IL-33 but did identify increased intestinal IL-25 and TSLP (thymic stromal lymphopoietin) that similarly correspond with oviposition timing (29). As IL-33, IL-25, and TSLP have been suggested to provide functional redundancy (80), the differences between *Schistosoma* species may be solely due to evolutionary preferences. The same trends with cytokines in this dataset appear to be consistent with cases of *S. mansoni* infections in humans where blood IL-33 and ST2 showed reduced mRNA in peripheral blood cells yet elevated IL-4, IL-6, and IL-10 measured via cytometric beads (81). Additionally, the cytokine responses are temporal and trend together; previously, it was demonstrated that GM-CSF, TNF-α, IL-13, IL-2, IL-4, IL-17, IFN-γ, IL-5, were all increased in the serum of C57BL/6J (relatively less IL-4 vs. other studies) that all corresponded to the timing of adult pairing and oviposition (82). In terms of the trend, these data agree with the cytokine profile identified here (**Figure 5**).

The complete blood count and leukocyte differential obtained from IDEXX BioAnalytics (**Tables 2**, **3**) has limitations in the that it includes a single chronically infected animal; however, the reference dataset is robust (n=7). Notably, the panel includes expanded hematology parameters such as reticulocytes that may be of interest to the field. The case study of the 19 week post-infection mouse can be insightful as it may provide a snapshot of a late-stage chronic infection, whereas many studies (e.g. worm burden reduction) are typically performed within 10 weeks post-infection. However, one possibility which cannot be eliminated is that this particular mouse may have achieved a reduced worm burden, through natural means or otherwise, despite displaying notably unwell behavior and appearance. Of note, the previously mentioned absence of basophils in circulation (**Table 3**), yet increased abundance in the splenocytes breaks the trend compared to neutrophils and eosinophils which were increased in both the the adult-stage infected spleen (7 weeks PI; **Table 1**) and in the circulation of a late-stage chronic infection (19 weeks PI; **Table 3**). This discrepancy between elevated basophils in the infected spleen (**Table 1**) but absence in the peripheral blood (**Table 3**) could indicate migration patterns or tissue-specific roles that would require further studies to delineate. Additionally, it remains possible that this change could be due to differences between infection durations, the limited sample size of the study, or instrument limits of detection; thus, these findings will need to be validated for reproducibility.

More broadly, relatively less is known about the role of granuolytes in late-stage infection, although eosinophils (predominantly), neutrophils, and macrophages are consistently found in tissue granulomas (83). The role eosinophils play in the late-stage of granulomatous infection is under reconsideration, with these cells possibly serving in an immune modulatory role, as opposed to a solely worm- or egg-targeting purpose (84). Similarly, the basophil contribution to schistosomiasis is poorly understood; however, it has been demonstrated previously that basophil depletion decreases the size of both lung and liver granulomatous lesions, providing a possible link to pathogenesis (68). These data confirm previous findings of splenic basophilia, which was similarly observed in *S. mansoni*-infected female BALB/c mice (68). Over time, the outer perimeter of granulomas appears to become infused with B lymphocytes, possibly in support of antibody production against the highly antigenic eggs, which generate a Th2 response in the host; however, these may also play a regulatory role (57). Altogether, the immune system appears to carefully balance inflammation and prevention of disease progression in *S. mansoni* infections, possibly leveraging granulocytes in this effort, although more work is needed to evaluate this (85).

Our current findings advance our understanding of an outbred *S. mansoni* mouse model frequently used for therapeutic and vaccine development. Advancing our understanding of the unique and complex immune response facilitated by human-relevant *Schistosoma* species may lead to novel therapeutic targets and vaccine opportunities that would enable us to reduce the global health burden caused by schistosomiasis which remains a top neglected infectious disease in the developing world (86).

## Data Availability

The original contributions presented in the study are included in the article/supplementary material. Further inquiries can be directed to the corresponding author.
